# Early prediction of thyroid capsule invasion in papillary microcarcinoma using ultrasound-based deep learning models: a retrospective multicenter study

**DOI:** 10.1186/s13244-025-02132-0

**Published:** 2025-11-27

**Authors:** Lin Sui, Bojian Feng, Xiayi Chen, Zhiyan Jin, Xinying Zhu, Tian Jiang, Yuqi Yan, Yahan Zhou, Chen Chen, Jincao Yao, Min Lai, Lujiao Lv, Yifan Wang, Liping Wang, Cong Li, Lina Feng, Wenwen Yue, Daizhang Yu, Kaiyuan Shi, Vicky Yang Wang, Yang Zhang, Dong Xu

**Affiliations:** 1 Department of Diagnostic Ultrasound Imaging & Interventional Therapy, Zhejiang Cancer Hospital, Hangzhou Institute of Medicine (HIM), Chinese Academy of Sciences, Hangzhou, China; 2 Center of Intelligent Diagnosis and Therapy (Taizhou), Hangzhou Institute of Medicine(HIM), Chinese Academy of Sciences, Taizhou, China; 3 Wenling Institute of Big Data and Artificial Intelligence in Medicine, Taizhou, China; 4 Taizhou Key Laboratory of Minimally Invasive Interventional Therapy & Artificial Intelligence, Taizhou Campus of Zhejiang Cancer Hospital (Taizhou Cancer Hospital), Taizhou, China; 5 Research Center of Interventional Medicine and Engineering, Hangzhou Institute of Medicine (HIM), Chinese Academy of Sciences, Hangzhou, China; 6 Postgraduate training base Alliance of Wenzhou Medical University (Zhejiang Cancer Hospital), Hangzhou, China; 7 Cancer Center, Department of Ultrasound Medicine, Zhejiang Provincial People’s Hospital (Affiliated People’s Hospital), Hangzhou Medical College, Hangzhou, China; 8 Department of Ultrasound, The First Hospital of Jiaxing & The Affiliated Hospitalof Jiaxing University, Jiaxing, China; 9 Center of Minimally Invasive Treatment for Tumor, Department of Medical Ultrasound, Shanghai Tenth People’s Hospital, School of Medicine, Tongji University, Shanghai, China; 10 Shanghai Engineering Research Center of Ultrasound Diagnosis and Treatment, National Clinical Research Center for Interventional Medicine, Shanghai, China; 11 Jinhua People’s Hospital, Jinhua, China; 12 Wenzhou Medical University, Wenzhou, China

**Keywords:** Papillary thyroid microcarcinoma, Ultrasound, Thyroid capsule invasion, Deep learning, Radiomics feature

## Abstract

**Objective:**

Thyroid capsule invasion (TCI) predicts early progression in papillary thyroid microcarcinoma (PTMC). This study aimed to develop an integrated model that combines handcrafted peri-tumoral radiomics features with deep learning (DL)-derived intra-tumoral features for accurate early prediction of TCI, to support clinical decision-making.

**Materials and methods:**

Retrospective data from 964 patients with 964 pathologically confirmed PTMC lesions across three centers were collected. Radiomics features were extracted from multiple peri-tumoral regions, and the optimal peri-tumor region with the best radiomics features was selected using a support vector machine (SVM). The selected radiomics features were then combined with intra-tumoral DL features extracted from the tumors before being fed into four different DL models for training and validation. Performance was validated on the internal (*n* = 177) and external (*n* = 84) test sets. Six radiologists (senior/attending/junior) assessed TCI with/without DL assistance.

**Results:**

The radiomics features, which achieved the best diagnostic performance with an AUC of 0.795 using SVM, were extracted from the peri-tumor region with 30% expansion from the original tumor. By further combining these radiomics features with intra-tumoral DL features, four different DL models were established to identify TCI in PTMC. Swin-Transformer achieved superior performance (internal AUC: 0.923; external AUC: 0.892). With DL model assistance, the AUCs of six radiologists significantly improved, for example, from 0.720 to 0.796 and from 0.725 to 0.790 for senior radiologists, and similar gains were observed for attending and junior radiologists.

**Conclusions:**

As an effective clinical assistive tool,  this integrated model can provide TCI identification with high level of accuracy. With its ability to enhance radiologists’ diagnostic performance, it supports early PTMC risk stratification and personalized intervention.

**Critical relevance statement:**

This retrospective multicenter study establishes an integrated model for identifying TCI in PTMC. The model significantly enhances radiologists’ diagnostic precision across multiple experience levels, supporting early clinical decision-making for optimized intervention strategies.

**Key Points:**

- Accurate prediction of TCI facilitates early assessment of PTMC progression and guides subsequent individualized clinical management.
- DL significantly improves the predictive performance for TCI.
- DL effectively assists radiologists in TCI diagnosis.

**Graphical Abstract:**

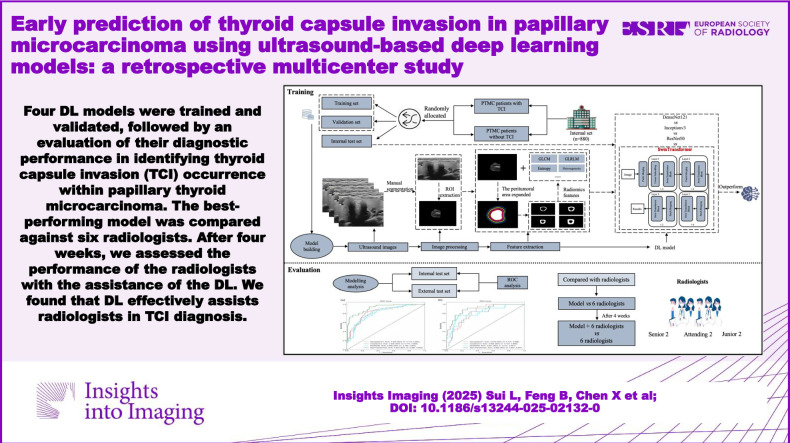

## Introduction

Papillary thyroid microcarcinoma (PTMC) refers to papillary thyroid carcinoma with a tumor size ≤ 10 mm, and it is one of the main contributors to the increasing incidence of thyroid cancer [1]. Over the past few decades, the global incidence of PTMC has been on the rise while the mortality rate remains stable, suggesting potential overtreatment of PTMC patients [2]. To address this concern, the management of PTMC has shifted from immediate surgery to more conservative approaches, such as active surveillance (AS) using neck ultrasound (US) examinations [3–5]. However, approximately 5% to 10% of PTMC patients may experience rapid disease progression, which could necessitate more extensive surgery if delayed by AS [6**–**8]. Thyroid capsule invasion (TCI) typically occurs in the early stages, where cancerous tissue infiltrates the continuous fibrous thyroid capsule, without extending into surrounding soft tissues or the sternothyroid muscle, signaling disease advancement. TCI is a prerequisite for significant extrathyroidal extension (sETE) [9]. According to the 8th edition of the American Joint Committee on Cancer (AJCC) TNM staging system for thyroid cancer [10], the presence of TCI alone does not alter the T stage. However, sETE upgrades the tumor from T1–T2 (confined to the thyroid) to T3–T4 (invading beyond the thyroid gland). Numerous studies [11–13] indicate that TCI is an independent risk factor for central and lateral lymph node metastasis (LNM). Early research [14, 15] also suggests that TCI increases the risk of poor prognosis. Early and accurate identification of TCI in PTMC patients is critical for assessing tumor progression. Additionally, preoperative TCI determination can guide surgeons in performing comprehensive intraoperative lymph node evaluations, given the low sensitivity of US in detecting central compartment LNM [16**]**.

Compared to MRI and CT, US offers advantages such as non-ionizing radiation, high portability, and real-time imaging, making it the most commonly used non-invasive imaging modality for early identification of thyroid disease [17]. Currently, the gold standard for TCI diagnosis remains postoperative pathological examination, which is invasive and unsuitable for early detection. Consequently, early TCI identification remains challenging due to the lack of standardized imaging criteria for TCI—for instance, TI-RADS includes margin characteristics but does not explicitly address capsular invasion—leading to considerable variability in radiologists’ interpretation and frequent underreporting in clinical practice[18, 19]. Thus, there is an urgent need for novel diagnostic tools to aid clinicians in early TCI detection and proactive tumor progression assessment.

With the rapid advancement of artificial intelligence (AI) technology, computer algorithms based on deep learning (DL) have made significant breakthroughs in the field of diagnostic medicine [20–22]. With its capabilities of extracting superior image features, AI has the potential to provide richer and more precise feature information to aid thyroid US diagnosis. By leveraging detailed feature extraction techniques, AI models can analyze thyroid US images and provide valuable insights for early cancer detection. The combination of DL algorithms and medical imaging data enables the development of robust diagnostic systems that can augment the expertise of radiologists and contribute to more timely and accurate diagnoses. Currently, there is considerable research on the classification of benign and malignant thyroid nodules, but its application for predicting specific pathological features like TCI—a key indicator of early progression—remains largely unexplored, especially PTMC. This lack of analysis can affect the early assessment of malignant tumor progression and subsequent personalized clinical management.

This study hypothesized that in PTMC cases with TCI, where cancerous tissue infiltrates the thyroid capsule, there may be crucial information around the nodules that are present on US images but not visible to the human naked eye. To this end, we developed a DL framework that integrates handcrafted radiomic characteristics from peri-tumoral regions with deep intra-tumoral features extracted using convolutional neural networks. This model was trained using multi-center US data to enable early and accurate identification of TCI in PTMC. The performance of the developed model was compared with that of radiologists of different experience levels in diagnosing TCI, hence evaluating the efficacy of the DL model in assisting radiologists with early and precise TCI diagnosis.

## Materials and methods

### Clinical data

This study was approved by the local institutional ethics committees of Zhejiang Cancer Hospital (IRB-2024-494 (IIT)), Taizhou Cancer Hospital (IRB-2024-056-IIT), and Shanghai Tenth People’s Hospital (IRB-2019-010-01), and the requirement for informed consent was waived.

**Fig. 1:**
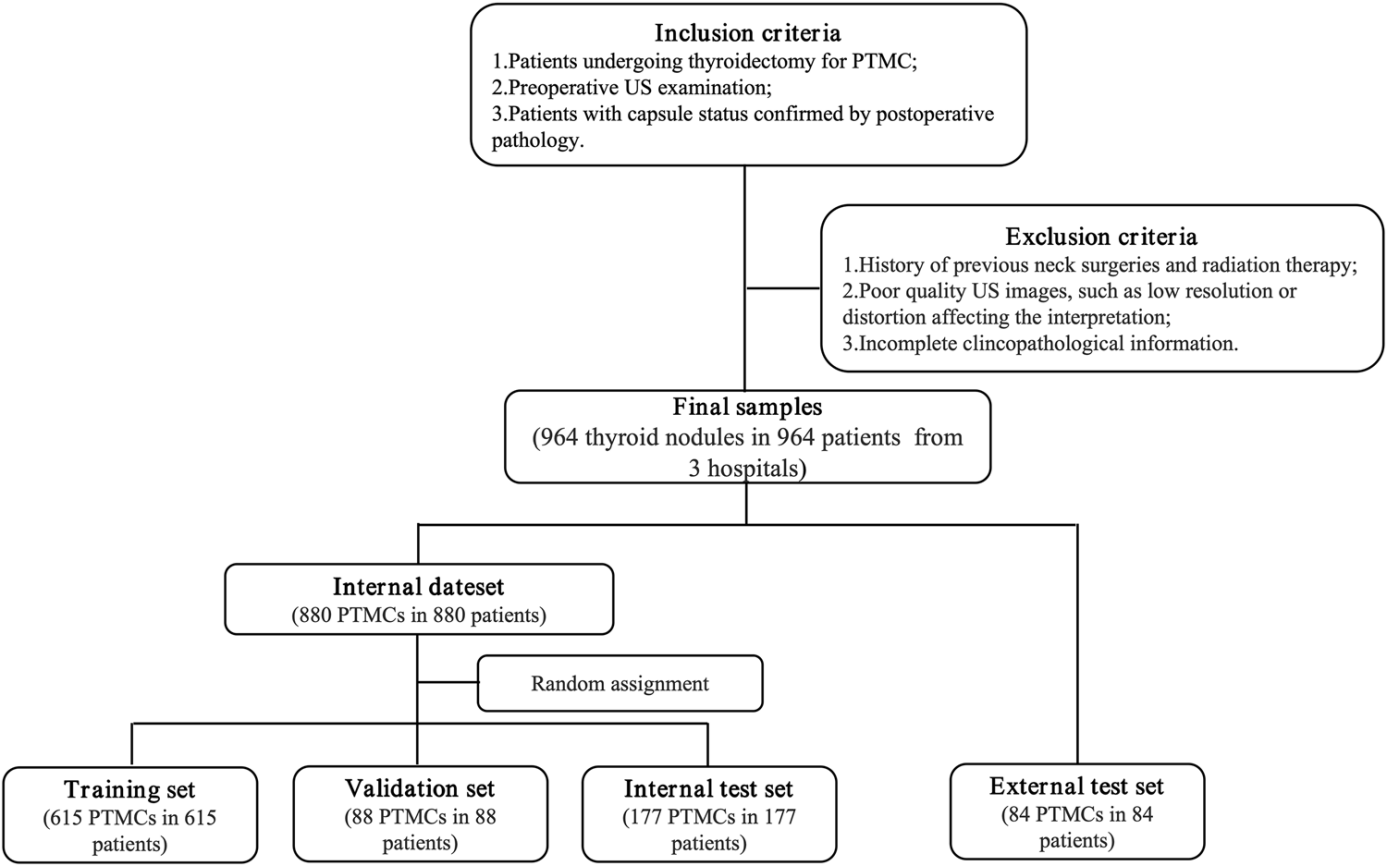
Patient recruitment flowchart with inclusion and exclusion criteria

**Fig. 2:**
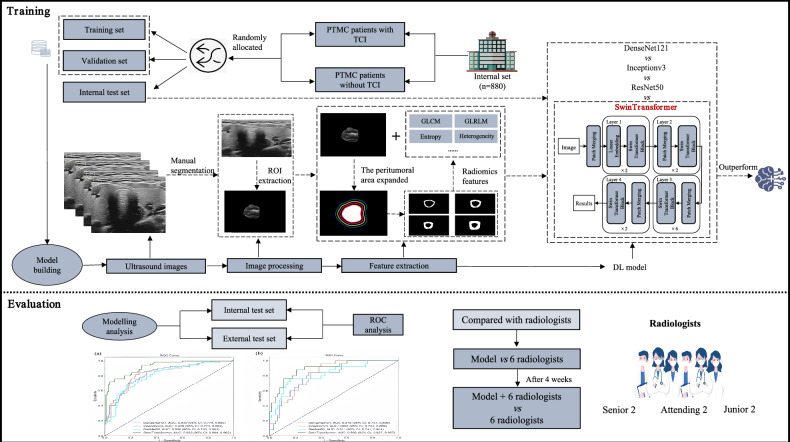
Overview of the study design. We conducted training and validation on four DL models, followed by an evaluation of their diagnostic performance in identifying TCI occurrence within PTMC on internal and external test sets. The best-performing DL model was selected and compared against the diagnostic performance of six radiologists, comprising two juniors, two attendings, and two senior radiologists. After four weeks, we assessed the performance of the six radiologists with the assistance of the DL model to determine whether it could enhance their diagnostic capabilities

A total of 964 lesions in 964 patients with PTMC were included in this retrospective multicenter study (Fig. 1). Specific details are presented in the Supplementary Materials. Within the internal dataset, the lesions were randomly divided into a training set (*n* = 615), a validation set (*n* = 88), and an internal test set (*n* = 177) using a ratio of 7:1:2. The external test set comprised 84 lesions. Figure 2 provides an overview of the study design.

### Data preprocessing

In this study, we first de-identified patient information from all collected raw DICOM images. Details of the collection of US and clinicopathological data are in the Supplementary Materials. For patients with multiple PTMC lesions in the same thyroid lobe, we matched the size and location of the target nodule from US images with the corresponding pathology results. We selected the nodules with the presence of TCI and/or the largest lesion for delineation. Additionally, to objectively assess the minimum nodule-to-capsule distance and the nodule-capsule contact length, the thyroid capsule was also delineated. A radiologist with five years of US imaging experience delineated all US images, which included two images per patient. The lesion boundaries and thyroid capsule contours were delineated using the labelme software and converted to binary masks using custom code written in Python. US characteristics, including the minimum distance from the nodule to the thyroid capsule, extent of capsule contact, nodule perimeter, and presence of capsular protrusion, were independently assessed and interpreted by the same radiologist. For capsular contact of the nodule on US, the assessment criterion is defined as follows: on the US image, there is no intervening thyroid tissue between the nodule and the posterior thyroid capsule, i.e., the two are directly adjacent. The assessment criterion for protrusion of the nodule on US is as follows: a typical manifestation of thyroid nodules invading or compressing the thyroid capsule, and the specific judgment basis is that the nodule causes outward bulging of the thyroid contour, with the bulging range exceeding the normal anatomical margin of the thyroid (i.e., the nodule breaks through the normal contour boundary of the thyroid itself and presents an “outward convex” morphology). All annotations and sonographic features underwent final review by a second board-certified radiologist with over 10 years of specialized experience in thyroid imaging. Finally, the lesion with TCI was labeled as “1” and the one without TCI was labeled as “0” in our analysis. After delineation, the regions of interest (ROIs) along with the TCI labels were exported to JSON format. Further processing of the images is in the Supplementary Materials.

### Extraction of radiomics features and establishment of DL models

We developed an integrated model that combines handcrafted radiomics features with DL-based representation learning for assessing TCI of PTMC. The details of extracting radiomics features are presented in the Supplementary Materials. After extracting radiomics features from the peri-tumor regions, we obtained the tumor boundary outlined by the radiologists and used this to extract the intra-tumoral features from the US images. Subsequently, the pixels within the tumoral area were normalized by subtracting the mean and dividing by the variance, and then resized to a uniform size of 224 × 224. To augment the data to six times its original volume, we employ methods such as random translation, random rotation, random cropping, random scaling, and random Gaussian noise. The handcrafted peri-tumoral radiomics features (*n* = 32) and the intra-tumoral image patches were jointly fed into a DL network. This combined approach aims to improve TCI diagnosis in PTMC by leveraging both engineered peri-tumoral characteristics and data-driven intra-tumoral features learned by the network. We selected four DL networks as the backbone networks of the model, including DenseNet-121, InceptionV3, ResNet-50, and SwinTransformer. The SwinTransformer-based classification model is a neural network model built using the self-attention mechanism. In our implementation, the Swin Transformer consisted of multiple encoder blocks followed by a classification head using a fully connected layer. Furthermore, DenseNet-121 mitigates the vanishing gradient problem with dense connections, InceptionV3 uses Inception modules to enhance perception, and ResNet-50 addresses the gradient issue through residual learning.

### Performance evaluation

In this study, a total of 84 cases of PTMC from two centers were collected as the external test set. The optimal model was compared with the diagnostic results of TCI by radiologists with varying years of experience on the external test set. This model was used to determine the probability of TCI on US images. The model’s performance was evaluated through receiver operating characteristic (ROC) curve analysis.

Additionally, the diagnostic performances of six radiologists were compared, including two senior radiologists, two attending radiologists, and two junior radiologists. Unaware of any clinical history, original clinical records, and pathological assessment results other than the fact that all nodules were PTMC, these six radiologists independently evaluated the TCI status of PTMC in the test dataset. They were required to diagnose whether PTMC had TCI two times, once without model assistance and once with model assistance, and with at least a two-week interval between the two diagnoses for all radiologists.

### Statistical analysis

For the baseline characteristics of the patients (Table 1), statistical analysis was conducted using SPSS 25.0 (IBM). The Shapiro–Wilk test was employed to assess the normality of data distribution. Continuous variables were presented as mean ± standard deviation (SD) or median (interquartile range), and comparisons between two groups were made using the independent samples *t*-test or Mann–Whitney *U*-test. Categorical variables were represented as frequencies and percentages, and comparisons between groups were conducted using the chi-square test or Fisher’s exact test.

**Table 1:** Baseline patient characteristics

Characteristics	Internal dataset (*n* = 880)			External test set (*n* = 84)		
	TCI (*n* = 314)	Non-TCI (*n* = 566)	*p* value	TCI (*n* = 23)	Non-TCI (*n* = 61)	*p* value
Age (years)^*^			**0.044**			0.414
Age ≥ 55	88 (28.03%)	127 (22.44%)		8 (34.78%)	15 (24.59%)	
Age < 55	226 (71.97%)	439 (77.56%)		15 (65.22%)	46 (75.41%)	
Sex			0.981			0.381
Female	236 (75.16%)	425 (75.09%)		16 (69.57%)	48 (78.69%)	
Male	78 (24.84%)	141 (24.91%)		7 (30.43%)	13 (21.31%)	
Size (mm)^*^			0.079			**0.030**
Size > 5	153 (48.73%)	241 (42.58%)		17 (73.91%)	29 (47.54%)	
Size ≤ 5	161 (51.27%)	325 (57.42%)		6 (26.09%)	32 (52.46%)	
Location of nodules			0.346			0.386
Left lobe	132 (42.04%)	257 (45.41%)		9 (39.13%)	33 (54.10%)	
Right lobe	164 (52.23%)	287 (50.71%)		13 (56.52%)	25 (40.98%)	
Isthmus	18 (5.73%)	22 (3.89%)		1 (4.35%)	3 (4.92%)	
TI-RADS scores			0.337			0.729
4	181 (57.64%)	345 (60.95%)		16 (69.57%)	40 (65.57%)	
5	133 (42.36%)	221 (39.05%)		7 (30.43%)	21 (34.43%)	
Capsular contact of the nodule on US			**< 0.001**			0.126
Absence	206 (65.61%)	473 (83.57%)		16 (69.57%)	53 (86.89%)	
Presence	108 (34.39%)	93 (16.43%)		7 (30.43%)	8 (13.11%)	
MNDR	0.008 (0.000. 0.029)	0.033 (0.009, 0.084)	**< 0.001**	0.012 (0.000, 0.025)	0.049 (0.008, 0.100)	**0.004**
NCCLR	0.000 (0.000, 0.031)	0.000 (0.000, 0.000)	**< 0.001**	0.000 (0.000, 0.016)	0.000 (0.000, 0.000)	0.071
Protrusion			**< 0.001**			0.796
Absence	268 (85.35%)	530 (93.64%)		20 (86.96%)	56 (91.80%)	
Presence	46 (14.65%)	36 (6.36%)		3 (13.04%)	5 (8.20%)	
LNM			0.019			0.084
Absence	199 (63.38%)	402 (71.02%)		12 (52.17%)	44 (72.13%)	
Presence	115 (36.62%)	164 (28.98%)		11 (48.83%)	17 (27.87%)	

Unless indicated otherwise, all other data are numbers with percentages in parentheses

*TCI* thyroid capsule invasion, *MNDR* the minimum nodule-to-capsule distance to perimeter ratio, *NCCLR* the nodule-capsule contact length to perimeter ratio

^*^Data are expressed as median with SD; *p* values were calculated from comparisons between the TCI and non-TCI groups using the chi-square test or Fisher’s exact test, with bold type indicating statistical significance (*p* < 0.05)

The model established in this study was built using the DL framework PyTorch 1.9.0, based on Python 3.8. Python was utilized for plotting ROC curves and calculating model performance indicators. Area under the curve (AUC) comparisons were carried out via the DeLong test. Statistical significance was set at *p* < 0.05.

## Results

### Baseline characteristics

This study included a total of 964 subjects, with 880 cases constituting the internal dataset (314 cases in the TCI group and 566 cases in the non-TCI group) and 84 cases forming the external test set (23 cases in the TCI group and 61 cases in the non-TCI group). In the internal dataset, the proportion of patients aged ≥ 55 years was significantly higher in the TCI group compared to the non-TCI group (28.03% vs 22.44%, *p *= 0.044). LNM was also more frequent in the TCI group (36.62% vs 28.98%, *p* = 0.019). Additionally, the presence of capsular contact on US was significantly associated with TCI (34.39% vs 16.43%, *p* < 0.001). In contrast, no significant differences were observed between the TCI and non-TCI groups in terms of sex distribution, nodule location, or TI-RADS scores. Specifically, TI-RADS 5 nodules were observed in 42.36% of TCI patients and 39.05% of non-TCI patients (*p* = 0.337). Within the external test set, a significantly larger proportion of patients in the TCI group had nodules larger than 5 mm (73.91% vs 47.54%, *p* = 0.030). LNM was more common in the TCI group (47.83% vs 27.87%, *p* = 0.084), though this difference did not reach statistical significance. The distribution of TI-RADS scores remained comparable between the two groups (TI-RADS 4: 69.57% vs 65.57%; TI-RADS 5: 30.43% vs 34.43%, *p* = 0.729). Further quantitative analysis is presented in the Supplementary Materials.

### Performance of the DL model

To investigate the imaging features surrounding the tumor regions, we evaluated radiomics features within different peri-tumor regions (e.g., 10–50%). Analysis revealed that radiomics features extracted from the 30% peri-tumor region (i.e., 1.3 times the size of the original tumor), the diagnostic performance of support vector machine (SVM) reached its maximum, with an AUC of 0.795 (Table S4 and Fig. S1). Consequently, we input the radiomic features extracted in this peri-tumor region, along with the intra-tumoral ROI from the US images, into four DL networks (DenseNet121, InceptionV3, ResNet50, and SwinTransformer) to determine the best model for detecting TCI.

**Fig. 3:**
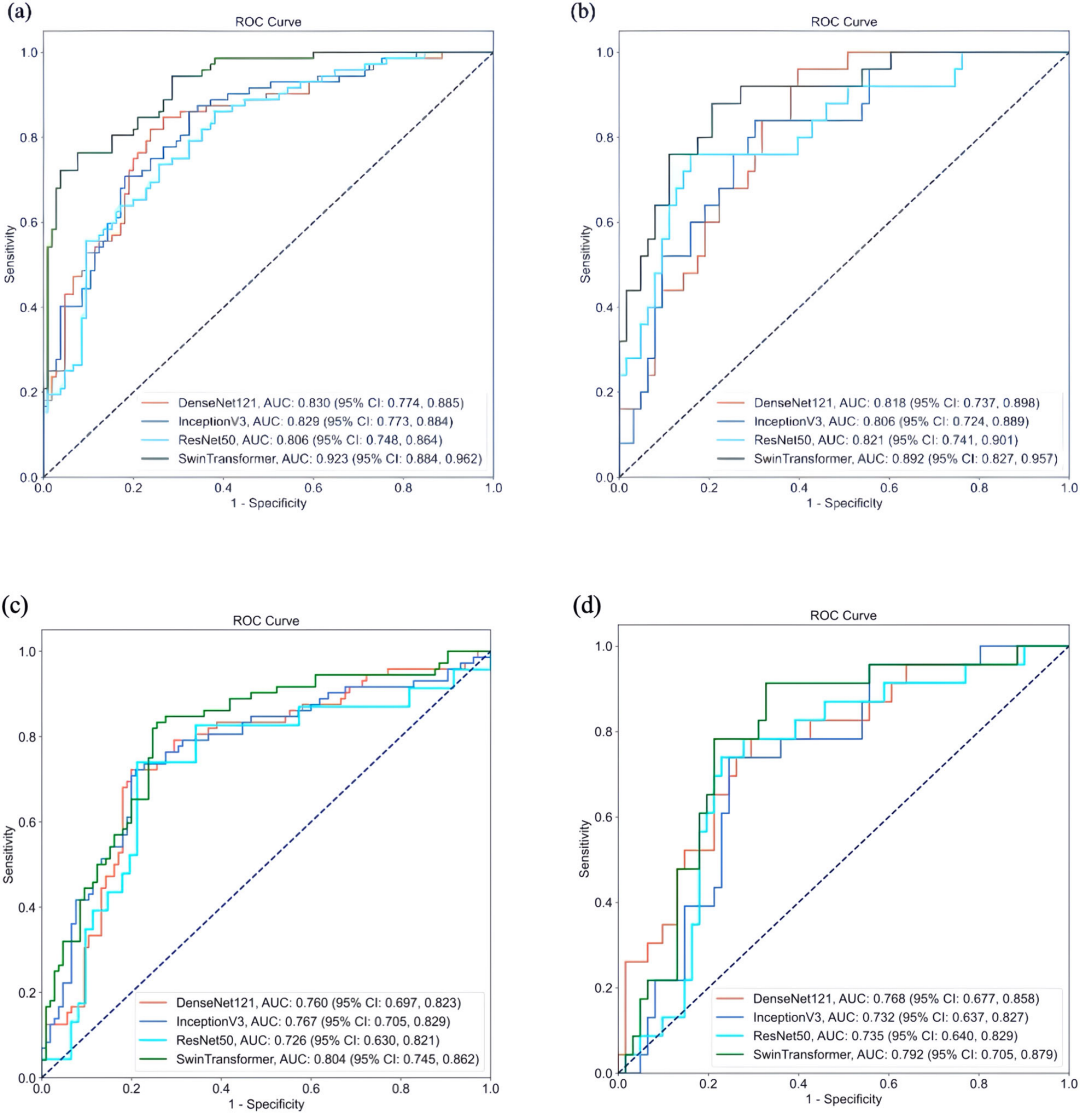
ROC curves of DL models. **a**, **b** represent the ROC curves of the internal test set and external test set, respectively, when peri-tumor radiomics features were combined with intra-tumor features to serve as input. Similarly, **c**, **d** represent the ROC curves of the internal test set and external test set, respectively, when intra-tumor features served as the sole input of the DL models

**Fig. 4:**
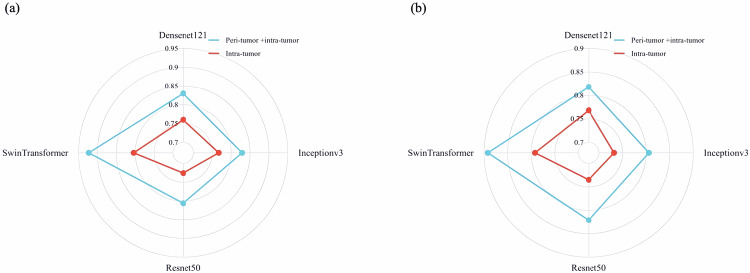
Radar maps illustrating the changes in AUC values for all four DL models using two inputs: intra-tumor and peri-tumor combined with intra-tumor. **a** Performance comparison based on the internal test set. **b** Performance comparison based on the external test set

In the internal test set (Table 2 and Fig. 3a), the Swin Transformer exhibited superior performance with an AUC of 0.923, sensitivity of 0.847, specificity of 0.771, and accuracy of 0.802, outperforming the other three DL models. In the external test set (Table 3 and Fig. 3b), the AUC for DenseNet121, InceptionV3, ResNet50, and SwinTransformer were 0.818, 0.806, 0.821, and 0.892, respectively, with SwinTransformer demonstrating the best performance. To demonstrate the utility of integrating peri-tumoral radiomics features, we trained four DL models using only intra-tumoral features for comparison. The results showed that SwinTransformer outperformed the other models when only intra-tumoral features were used as input. Furthermore, incorporating peri-tumoral radiomics features significantly improved its performance. In the internal test set (Table 3 and Fig. 3c), the AUC improved from 0.804 to 0.923, and in the external test set (Table 3 and Fig. 3d), the AUC increased from 0.792 to 0.892. Figure 4 illustrates the change in AUC values across all four models when comparing intra-tumoral input alone vs combined intra- and peri-tumoral features. Test results for different subgroups are presented in the Supplementary Materials.

**Table 2:** Performance of the DL model in the internal test set

	Densenet121		Inceptionv3		Resnet50		SwinTransformer	
Input	Peri-tumor + intra-tumor	Intra-tumor	Peri-tumor + intra-tumor	Intra-tumor	Peri-tumor + Intra-tumor	Intra-tumor	Peri-tumor + intra-tumor	Intra-tumor
Accuracy (95% CI)	0.780 (0.719, 0.841)	0.763 (0.700, 0.825)	0.740 (0.676, 0.805)	0.751 (0.688, 0.815)	0.718 (0.651, 0.784)	0.750 (0.657, 0.843)	0.802 (0.744, 0.861)	0.780 (0.719, 0.841)
Sensitivity (95% CI)	0.792 (0.698, 0.885)	0.722 (0.619, 0.826)	0.750 (0.650, 0.850)	0.736 (0.634, 0.838)	0.750 (0.650, 0.850)	0.739 (0.560, 0.919)	0.847 (0.764, 0.930)	0.819 (0.731, 0.908)
Specificity (95% CI)	0.771 (0.691, 0.852)	0.790 (0.713, 0.868)	0.733 (0.649, 0.818)	0.762 (0.680, 0.843)	0.695 (0.607, 0.783)	0.754 (0.646, 0.862)	0.771 (0.691, 0.852)	0.752 (0.670, 0.835)
PPV (95% CI)	0.704 (0.604, 0.803)	0.703 (0.599, 0.807)	0.659 (0.556, 0.761)	0.679 (0.576, 0.783)	0.628 (0.526, 0.730)	0.531 (0.358, 0.704)	0.718 (0.622, 0.813)	0.694 (0.596, 0.792)
NPV (95% CI)	0.844 (0.771, 0.916)	0.806 (0.729, 0.882)	0.811 (0.732, 0.889)	0.808 (0.731, 0.886)	0.802 (0.720, 0.884)	0.885 (0.798, 0.971)	0.880 (0.814, 0.947)	0.859 (0.788, 0.930)
AUC (95% CI)	0.830 (0.774, 0.885)	0.760 (0.697, 0.823)	0.829 (0.773, 0.884)	0.767 (0.705, 0.829)	0.806 (0.748, 0.864)	0.726 (0.630, 0.821)	0.923 (0.884, 0.962)	0.804 (0.745, 0.862)
K (Kappa)	0.745	0.712	0.701	0.707	0.684	0.618	0.777	0.752

*CI* confidence interval, *PPV* positive prediction value, *NPV* negative prediction value, *AUC* area under the curve

**Table 3:** Performance of the DL model in the external test set

	Densenet121		Inceptionv3		Resnet50		SwinTransformer	
Input	Peri-tumor +intra-tumor	Intra-tumor	Peri-tumor +intra-tumor	Intra-tumor	Peri-tumor +ntra-tumor	Intra-tumor	Peri-tumor +intra-tumor	Intra-tumor
Accuracy (95% CI)	0.716 (0.622, 0.810)	0.714 (0.618, 0.811)	0.739 (0.647, 0.830)	0.726 (0.631, 0.822)	0.818 (0.738, 0.899)	0.762 (0.671, 0.853)	0.795 (0.711, 0.880)	0.786 (0.698, 0.873)
Sensitivity (95% CI)	0.800 (0.643, 0.957)	0.783 (0.614, 0.951)	0.720 (0.544, 0.896)	0.739 (0.560, 0.919)	0.760 (0.593, 0.927)	0.739 (0.560, 0.919)	0.800 (0.643, 0.957)	0.783 (0.614, 0.951)
Specificity (95% CI)	0.683 (0.568, 0.797)	0.689 (0.572, 0.805)	0.746 (0.639, 0.854)	0.721 (0.609, 0.834)	0.841 (0.751, 0.932)	0.770 (0.665, 0.876)	0.794 (0.694, 0.894)	0.787 (0.684, 0.890)
PPV (95% CI)	0.500 (0.345, 0.655)	0.486 (0.325, 0.648)	0.529 (0.362, 0.697)	0.500 (0.332, 0.668)	0.655 (0.482, 0.828)	0.548 (0.373, 0.724)	0.606 (0.439, 0.773)	0.581 (0.407, 0.754)
NPV (95% CI)	0.896 (0.809, 0.982)	0.894 (0.805, 0.982)	0.870 (0.781, 0.960)	0.880 (0.790, 0.970)	0.898 (0.821, 0.975)	0.887 (0.801, 0.972)	0.909 (0.833, 0.985)	0.906 (0.827, 0.984)
AUC (95% CI)	0.818 (0.737, 0.898)	0.768 (0.677, 0.858)	0.806 (0.724, 0.889)	0.732 (0.637, 0.858)	0.821 (0.741, 0.901)	0.735 (0.640, 0.829)	0.892 (0.827, 0.957)	0.792 (0.705, 0.879)
K (Kappa)	0.615	0.600	0.610	0.596	0.704	0.630	0.690	0.667

*CI* confidence interval, *PPV* positive prediction value, *NPV* negative prediction value, *AUC* area under the curve

### Diagnostic performance of the Radiologist and DL model-assisted diagnosis

We compared the TCI diagnostic performance of six radiologists with varying levels of experience against the best-performing DL model using the external test set. As shown in Table 4, the diagnostic performance of SwinTransformer was significantly better than that of the six radiologists (all *p* < 0.05), with an AUC of 0.892. The AUCs for the senior, attending, and junior radiologists were 0.720/0.725, 0.685/0.665, and 0.643/0.635, respectively.

**Table 4:** Performance of the DL model and six radiologists in the external test set

	Accuracy (95% CI)	Sensitivity (95% CI)	Specificity (95% CI)	PPV (95% CI)	NPV (95% CI)	AUC (95% CI)	*p* value
SwinTransformer	0.795 (0.711, 0.880)	0.800 (0.643, 0.957)	0.794 (0.694, 0.894	0.606 (0.439, 0.773)	0.909 (0.833, 0.985)	0.892 (0.827, 0.957)	
Radiologists alone							
Junior 1	0.679 (0.579, 0.778)	0.565 (0.363, 0.768)	0.721 (0.609, 0.834)	0.433 (0.256, 0.611)	0.815 (0.711, 0.918)	0.643 (0.541, 0.746)	0.003^*^
Junior 2	0.726 (0.631, 0.822)	0.435 (0.232, 0.637)	0.836 (0.743, 0.929)	0.500 (0.281, 0.719)	0.797 (0.698, 0.895)	0.635 (0.532, 0.738)	< 0.001^*^
Attending 1	0.798 (0.712, 0.884)	0.435 (0.232, 0.637)	0.934 (0.872, 0.997)	0.714 (0.478, 0.951)	0.814 (0.723, 0.905)	0.685 (0.585, 0.784)	0.002^*^
Attending 2	0.750 (0.657, 0.843)	0.478 (0.274, 0.682)	0.852 (0.763, 0.941)	0.550 (0.332, 0.768)	0.812 (0.717, 0.908)	0.665 (0.564, 0.766)	0.003^*^
Senior 1	0.750 (0.657, 0.843)	0.652 (0.458, 0.847)	0.787 (0.684, 0.890)	0.536 (0.351, 0.720)	0.857 (0.765, 0.949)	0.720 (0.623, 0.816)	0.032^*^
Senior 2	0.738 (0.644, 0.832)	0.696 (0.508, 0.884)	0.754 (0.646, 0.862)	0.516 (0.340, 0.692)	0.868 (0.777, 0.959)	0.725 (0.629, 0.820)	0.021^*^
Radiologists + AI							
Junior 1	0.738 (0.644, 0.832)	0.783 (0.614, 0.951)	0.721 (0.609, 0.834)	0.514 (0.349, 0.680)	0.898 (0.813, 0.983)	0.752 (0.660, 0.844)	0.087^^^
Junior 2	0.786 (0.698, 0.873)	0.435 (0.232, 0.637)	0.918 (0.849, 0.987)	0.536 (0.351, 0.720)	0.812 (0.719, 0.904)	0.676 (0.576, 0.776)	0.256^^^
Attending 1	0.845 (0.768, 0.923)	0.565 (0.363, 0.768)	0.951 (0.897, 1.005)	0.812 (0.621, 1.004)	0.853 (0.769, 0.937)	0.758 (0.666, 0.850)	0.264^^^
Attending 2	0.810 (0.726, 0.893)	0.739 (0.560, 0.919)	0.836 (0.743, 0.929)	0.630 (0.447, 0.812)	0.895 (0.815, 0.974)	0.788 (0.700, 0.875)	**0.012**^^^
Senior 1	0.821 (0.740, 0.903)	0.739 (0.560, 0.919)	0.852 (0.763, 0.941)	0.654 (0.471, 0.837)	0.897 (0.818, 0.975)	0.796 (0.710, 0.882)	0.191^^^
Senior 2	0.774 (0.684, 0.863)	0.826 (0.671, 0.981)	0.754 (0.646, 0.862)	0.559 (0.392, 0.726)	0.920 (0.845, 0.995)	0.790 (0.703, 0.877)	0.299^^^

*CI* confidence interval, *PPV* positive prediction value, *NPV* negative prediction value, *AUC* area under the curve, *AI* artificial intelligence

^*^p values refer to the AI system; the *p*-values were calculated based on the comparison of AUCs derived from the DeLong test, bold indicates statistically significant (*p* < 0.05)

^^^*p* values refer to radiologists alone; the *p*-values were calculated based on the comparison of AUCs derived from the DeLong test, bold indicates statistically significant (*p* < 0.05)

In addition to this, we analyzed the diagnostic performance of the six radiologists with the assistance of the best model. With model assistance, AUCs improved for all radiologists: 0.796 and 0.790 for the senior group, 0.758 and 0.788 for the attending group, and 0.752 and 0.676 for the junior group. One attending radiologist’s AUC increased significantly from 0.665 to 0.788 (*p* = 0.012). Figure S3 illustrates the changes in diagnostic performance metrics among 6 radiologists with and without model assistance. Details are shown in the Supplementary Materials.

### Visualization and auxiliary diagnosis function of the DL model

**Fig. 5:**
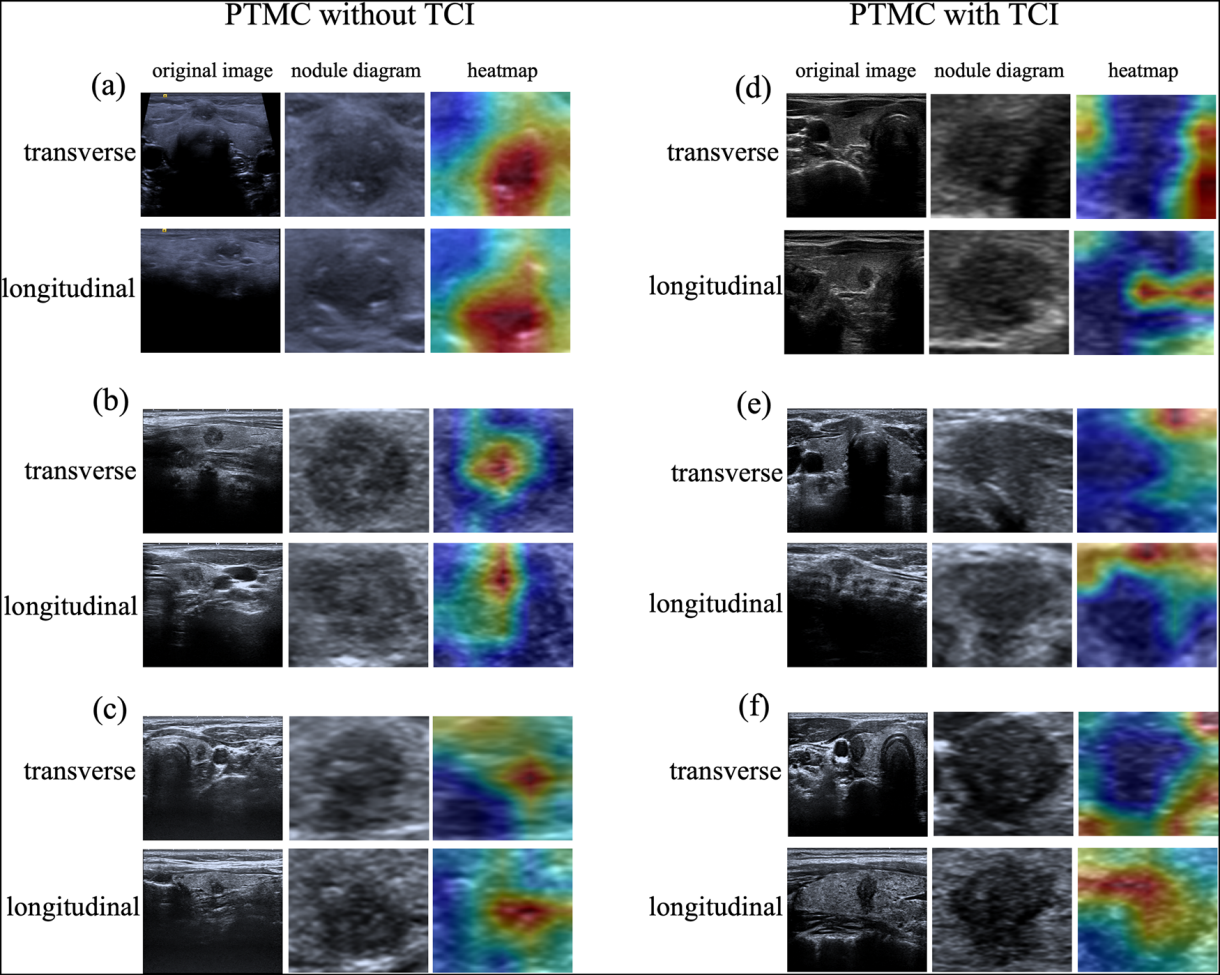
Characteristic heat maps. Representative PTMC cases  without TCI are shown  in panels **a**, **b**, and **c**, while cases with TCI are shown in panels **d**, **e** and **f**. Each case included both US transverse and longitudinal planes. Each displayed image is divided into three columns: original US image, cropped image to focus on nodules, and the heat map

We selected six representative cases to visualize the diagnostic outcome of the DL model (Fig. 5). Heatmaps generated through class activation mapping enhance the interpretability of the model. Different color distributions reflect the SwinTransformer model’s focus on predictive regions and image features within the US images. Regions highlighted in red provide more informative features during the prediction process. For cases (a), (b), and (c), which all represent PTMC without TCI, the heatmap attention is concentrated on the nodular area. Conversely, for cases (d), (e), and (f) showing PTMC with TCI, the heatmap’s attention is focused on specific regions close to the edges of the image. In addition, to elucidate the contributions of the 32 peritumoral radiomics features to the SVM model for TCI classification in both internal and external test sets, SHAP (SHapley Additive exPlanations) analysis was performed, with results visualized in Fig. S4 (internal test set) and Fig. S5 (external test set).

## Discussion

TCI, as an early indicator of tumor progression, is of great diagnostic significance for PTMC patients. In this study, we first applied SVM to determine the peri-region with the most prominent radiomic features. These were then combined with DL-derived intra-tumoral imaging features extracted via DL to explore the optimal DL model for predicting TCI in PTMC. We found that the radiomic features extracted from the peri-tumor region (i.e., 1.3 times the size of the original tumor) demonstrated the best diagnostic performance with an AUC of 0.795. The SwinTransformer demonstrated the optimal performance, with an AUC of 0.923 on the internal test set and an AUC of 0.892 on the external test set, outperforming the other three DL models. Additionally, the performance of the SwinTransformer model was significantly superior to that of radiologists at various experience levels. Furthermore, with model assistance, there was a certain degree of improvement in the AUC, accuracy, and sensitivity of radiologists at different experience levels. This study represents the first attempt to simultaneously incorporate peri-tumoral radiomics features alongside intra-tumoral features as inputs for DL models. The results demonstrate significant improvement in diagnosing TCI, aiming to better guide early clinical practices.

AS is a commonly adopted strategy in managing patients with PTMC. Careful risk stratification and appropriate patient selection are essential for successful AS implementation [8]. Since TCI is one of the important factors in tumor progression, selecting AS in patients with undetected TCI may increase the risk of progression to extrathyroidal extension (ETE) or LNM [11–13]. Therefore, early identification of TCI in PTMC remains an active clinical question. However, the assessment of TCI by the US largely depends on radiologists, and its accuracy varies among patients and observers. Previous studies [23–25] have shown that the accuracy of routine US assessment of TCI ranges from 66.1% to 81.9%. Jeong et al [26] retrospectively analyzed the US features of PTMC patients with posterior capsule invasion and their clinical significance. They found that the diagnostic performance of relevant US features in predicting posterior capsule invasion in PTMC patients was 71.3–75.1%, and PTMC patients with posterior minor TCI were more likely to have lymphovascular invasion and lateral neck LNM (OR = 2.636, 95% confidence interval (CI): 1.754, 3.963 and OR = 2.897, 95% CI: 1.069, 7.848). Additionally, they analyzed the interobserver consistency in assessing US features. The results showed moderate consistency among observers for abutment assessment (*K* = 0.705), while abutment degree according to tumor perimeter and protrusion assessment exhibited lower interobserver consistency (*K* = 0.553 and 0.287, respectively). Due to the absence of standardized US features for evaluating and diagnosing TCI, radiologists’ performance and consistency in diagnosing TCI are relatively low. Therefore, to mitigate subjective influences, AI was introduced to assist radiologists in early and accurate identification of TCI. In a previous study [27], based on CT imaging, a radiomics model predicted TCI in PTC. The model achieved an AUC of 0.820 in internal testing and 0.776 in external testing. In contrast, our study utilized US images, which are radiation-free, user-friendly, and provide high soft tissue resolution. Our study, based on the US, achieved better diagnostic performance compared to the previous study [27]. Moreover, unlike previous studies, our study considered features around the nodules and depicted the state of TCI with interpretable features. Analyzing the nodule itself in US images may overlook its relationship with the capsule. Therefore, we extracted and selected peri-tumoral radiomics features, which, together with intra-tumoral DL features from US images, were input into a DL model to identify PTMC’s TCI. Our best-trained model (SwinTransformer) demonstrated good performance in diagnosing TCI and exhibited favorable reproducibility and generalizability on external test sets.

In the external test set, the individual performance of TCI diagnosis by six radiologists was found to be unsatisfactory. The AUCs were 0.643 and 0.635 for junior radiologists, 0.685 and 0.665 for attending radiologists, and 0.720 and 0.725 for senior radiologists. This could partly be attributed to the fact that we only provided static US images to the six radiologists, whereas clinical US examination is a dynamic process. Additionally, the absence of standardized criteria for evaluating TCI means that radiologists heavily rely on their subjective judgment and past experiences, leading to potential misdiagnoses. Model assistance led to moderate improvements in radiologists’ diagnostic performance, suggesting its potential utility in supporting TCI diagnosis. However, most improvements were not statistically significant, possibly reflecting limited integration of model outputs into the diagnostic workflow. Perhaps increasing the model’s interpretability could enhance radiologists’ trust in it in the future.

This study has several limitations that warrant attention in future research. First, the analysis relied on a limited number of retrospectively collected static US images. Future prospective studies should incorporate dynamic US videos to develop more robust and optimized models. Second, the study was confined to three centers and did not account for variations in imaging equipment specifications. Thus, broader validation across multi-center cohorts and larger datasets is essential to assess the model’s generalizability, particularly when addressing potential variability arising from different US devices. Third, manual segmentation was employed for image analysis, which is labor-intensive and prone to inter-/intra-observer variability. Implementing automated segmentation algorithms in future work could enhance reproducibility while reducing radiologists’ workload. Fourth, the stratification of cases did not consider the degree of capsular invasion. Given that TCI precedes ETE, subsequent studies should categorize cases based on histopathologically confirmed invasion depth to refine diagnostic precision. Lastly, data on concomitant thyroid parenchymal disorders—such as Hashimoto’s thyroiditis or nodular goiter—were not systematically collected in this retrospective study. The absence of this clinical information may limit a comprehensive understanding of the imaging context. Future prospective studies will include standardized evaluation and reporting of background thyroid conditions to better assess their potential influence on model performance.

## Conclusion

In summary, the integrated model developed in this study—combining peri-tumoral radiomics with DL-derived intra-tumoral features—demonstrated high diagnostic accuracy in identifying TCI in PTMC. The model serves as an effective assistive tool that enhances diagnostic consistency across radiologists of varying experience levels. It provides a standardized, image-based assessment of TCI that helps reduce interpretive subjectivity. These capabilities contribute to more reliable early risk stratification and support personalized clinical decision-making for PTMC patients

## Supplementary information

Supplementary information

## Data Availability

The datasets used and/or analyzed during the current study are available from the corresponding author upon reasonable request.

## Abbreviations

AI: Artificial intelligence
AS: Active surveillance
AUC: Area under the curve
CI: Confidence interval
DL: Deep learning
ETE: Extrathyroidal extension
LNM: Lymph node metastasis
PTMC: Thyroid papillary microcarcinoma
ROC: Receiver operating characteristic
ROI: Region of interest
SVM: Support vector machine
TCI: Thyroid capsule invasion
US: Ultrasound
