# Supplementary material for: Early prediction of thyroid capsule invasion in papillary microcarcinoma using ultrasound-based deep learning models: a retrospective multicenter study
Source: Insights Imaging. 2025 Nov 27;16:265. doi: 10.1186/s13244-025-02132-0 (PMC12660598; doi:10.1186/s13244-025-02132-0)
Supplement: Supplementary file 1 — Supplementary information [file 13244_2025_2132_MOESM1_ESM.pdf]

Early Prediction of Thyroid Capsule Invasion in Papillary Microcarcinoma Using Ultrasound-Based Deep Learning Models: A Retrospective Multicenter Study

ELECTRONIC SUPPLEMENTARY MATERIAL

Inclusion and exclusion criteria

Patients with PTMC admitted to Zhejiang Cancer Hospital from January 2019 to March 2020 were assigned as the internal dataset. Patients with PTMC admitted to Shanghai Tenth People's Hospital from October 2022 to March 2023 and Taizhou Cancer Hospital from September 2021 to July 2023 were assigned as the external test set. All patients underwent initial thyroidectomy.The inclusion criteria are as follows: (1) patients undergoing thyroidectomy for PTMC; (2) preoperative US examination; (3) patients with capsule status confirmed by postoperative pathology. The exclusion criteria are as follows: (1) history of previous neck surgeries and radiation therapy; (2) poor quality US images, such as low resolution or distortion affecting the interpretation; (3) incomplete clinicopathological information. Additionally, for patients with multifocal thyroid lesions, the one with either presence of TCI or the largest lesion was selected for analysis.

Collection of US and clinicopathological dates

The thyroid US images were obtained from the following ultrasound devices: LOGIQ E9 (General Electric Healthcare), EPIQ7 and iU22 (Philips Healthcare), and Aplio 500 (Canon Medical Systems). All scans were performed using a linear array transducer with a broadband frequency range of 5-12 MHz. All US examinations were performed by the radiologists, and the corresponding US images were stored. Typically, two ultrasound images showing the lesion's maximum diameters in longitudinal and transverse planes were exported in DICOM format for subsequent analysis. Clinicopathological information, including gender, age, and capsule status, was retrieved from the electronic medical records system.

The details of image processing

To ensure objective quantification of nodule-capsule spatial relationships, two normalized metrics were adopted: 1) the minimum nodule-to-capsule distance to perimeter ratio (MNDR) and 2) the nodule-capsule contact length to perimeter ratio (NCCLR). MNDR quantifies the shortest Euclidean distance from the thyroid nodule boundary to the thyroid capsule, normalized by the nodule's perimeter. The nodule boundary is first delineated on the ultrasound image, and the thyroid capsule is identified either manually or via segmentation. The minimum distance from any point on the nodule contour to the nearest point on the capsule is measured. This minimum distance is then divided by the total perimeter of the nodule to obtain the MNDR. For NCCLR, this metric measures the length of the interface where the nodule is in direct contact with the thyroid capsule, relative to the total perimeter of the nodule. After segmenting both the nodule and the capsule, the contact length is determined by summing the length of the contiguous segments where the two boundaries are adjacent or touching. This value is then divided by the total nodule perimeter to obtain the normalized the results. This normalization strategy effectively minimizes confounding effects from nodule size heterogeneity, thereby enhancing the robustness and biological relevance of spatial parameter analyses.

Based on the calculated MNDR and NCCLR, nodules were categorized into two groups: presence of capsular contact on US and absence of capsular contact on US. Within the group exhibiting presence of capsular contact on US, protrusion was also an important predictor for TCI. Therefore, the same two radiologists (the one with 5 years of experience and the other with 10 years) interpreted the nodules in this group for prominence, with the more experienced radiologist reviewing the assessments.

Extraction of radiomics features

Initially, we extracted the thyroid nodule regions from US images based on manually annotated json files and outlined the nodules using OpenCV. The peri-tumoral region was defined by expanding the nodule ROI outward by 10% to 50% (in 10% increments), based on the radial distance from the nodule center, as shown in Figure 2. For the region enclosed by each of the expanded ROIs, we sequentially extracted radiomics features using the PyRadiomics package. These features included morphological features (e.g. size, shape), texture features (e.g. gray-level co-occurrence matrix, gray-level run-length matrix), intensity features (e.g. average gray value, standard deviation, histogram features), and peripheral features (e.g. edge smoothness, curvature). As a result, each patient had two US cross-sections, and 682 × 5 radiomics features were extracted from the 5 peri-tumor regions in each US cross-section. To reduce feature dimensionality and improve model training efficiency, we employed Principal Component Analysis (PCA) for radiomics feature dimensionality reduction. PCA projects high-dimensional data into a lower-dimensional space while preserving maximal variance. Following PCA, the 682 × 5 features were reduced to 32 × 5 per image. A support vector machine (SVM) was then trained to classify TCI based on features from each peri-tumoral region, and the region yielding the highest classification performance was selected. The SVM classifier identifies the optimal peri-tumoral region based on prediction performance (see Table S1 for feature extraction parameters).

Baseline characteristics

Further quantitative analysis showed that in the internal dataset, MNDR, NCCLR and protrusion differed significantly between the TCI and non-TCI groups ($p < 0.001$). In contrast, MNDR was statistically significant in the external test dataset (Table 1). To evaluate the predictive role of ultrasound-detected nodule contact with the thyroid capsule for TCI, subgroup analyses were conducted. Among non-contacting nodules, MNDR consistently differed between TCI and non-TCI groups across both internal and external datasets ($p < 0.05$) (Table S2). In contrast, NCCLR and protrusion were not statistically significant in either dataset ($p > 0.05$) (Table S3).

Performances of DL model

Additionally, the SwinTransformer model was evaluated across distinct subgroups (Supplementary Figure S2). Stratified by age, the model achieved AUCs of 0.838 (age < 55) and 0.949 (age ≥ 55) in the internal test set, with corresponding values of 0.815 and 0.918 in the external test set. For nodule location, AUCs were 0.919 (isthmus) and 1.000 (bilateral lobes) internally, and 0.889 and 0.833 externally. When stratified by nodule size, the model yielded AUCs of 0.979 (≤ 5 mm) and 0.866 (>5 mm) in the internal test set, compared to 0.836 and 0.895 in the external set. For capsular contact on ultrasound, the model achieved AUCs of 0.973 (absence) and 0.857 (presence) in the external dataset, and 0.927 and 0.914 in the internal set. Finally, under protrusion stratification, the model demonstrated internal AUCs of 0.921 (absence) and 0.898 (presence), and external values of 0.870 and 0.983.

Visualization and auxiliary diagnosis function of DL model

In Figure S1's beeswarm plot (panel A), each point represents an individual patient in the internal test set, with the horizontal position reflecting the SHAP value (indicating the feature's contribution to prediction in log-odds) and color denoting the original feature value (red = high, blue = low); features are ordered by overall importance from top to bottom. In the internal test set (Figure S4), the SHAP beeswarm plot (Left) shows that LoG_glcm_Imc2 and wavelet-L_firstorder_Entropy are the top-ranked key features. For LoG_glcm_Imc2, high values (red points) correspond to positive SHAP values, increasing the probability of TCI prediction, while low values (blue points) correspond to negative SHAP values, decreasing the prediction probability. In contrast, for wavelet-L_firstorder_Entropy, high values (red points) correspond to negative SHAP values, reducing the probability of TCI prediction, and low values (blue points) correspond to positive SHAP values, increasing the prediction probability. The bar plot (Right) further verifies their average importance, with taller bars indicating stable contributions across samples.

In the external test set (Figure S5), original_firstorder_Variance serves as the primary driving feature. The beeswarm plot (Left) reveals that high values of this feature (red points) correspond to negative SHAP values, decreasing the probability of TCI prediction, and low values (blue points) correspond to positive SHAP values, increasing the prediction probability. In the bar plot (Right), this feature has the tallest bar, confirming its stable and significant average contribution in the external test set and its most prominent impact on model predictions.

Table S1. Thirty-two radiomics features were extracted.

original_firstorder_RobustMeanAbsoluteDeviation
original_firstorder_Variance
original_glcm_ClusterTendency
original_gldm_SmallDependenceLowGrayLevelEmphasis
original_glszm_SizeZoneNonUniformityNormalized
LoG_glcm_DifferenceEntropy
LoG_glcm_Imc2
LoG_gldm_LargeDependenceEmphasis
LoG_glrlm_LongRunEmphasis
LoG_glrlm_ShortRunLowGrayLevelEmphasis
gradient_gldm_DependenceNonUniformity
gradient_glrlm_ShortRunEmphasis
gradient_glszm_LargeAreaHighGrayLevelEmphasis
gradient_glszm_ZonePercentage
gradient_glszm_ZoneEntropy
wavelet-H_firstorder_RobustMeanAbsoluteDeviation
wavelet-H_firstorder_RootMeanSquared
wavelet-H_ngtdm_Contrast
wavelet-H_glcm_SumEntropy
wavelet-L_firstorder_Entropy
wavelet-L_firstorder_Skewness

wavelet-L_glrlm_RunPercentage
wavelet-L_glszm_SizeZoneNonUniformity
wavelet-L_glszm_SmallAreaEmphasis
wavelet-L_glszm_ZoneVariance
LBP2D_glcm_SumEntropy
LBP2D_gldm_LargeDependenceHighGrayLevelEmphasis
LBP2D_glrlm_RunPercentage
LBP2D_glrlm_ShortRunHighGrayLevelEmphasis
LBP2D_glszm_SmallAreaHighGrayLevelEmphasis
LBP2D_glszm_ZoneVariance
LBP2D_gldm_DependenceVariance

Table S2. Ultrasound characteristics of nodules without contact with the thyroid capsule

Characteristics	Internal set (n=679)			External test set (n=69)		
	TCI (n=206)	non-TCI (n=473)	p value	TCI (n=16)	non-TCI (n=53)	p value
MNDR	0.022 (0.008,0.051)	0.049 (0.021, 0.099)	<0.001	0.018 (0.011, 0.037)	0.060 (0.018, 0.105)	0.022

TCI, thyroid capsule invasion; MNDR, the minimum nodule-to-capsule distance to perimeter ratio.

Table S3. Ultrasound characteristics of nodules in contact with the thyroid capsule

Characteristics	Internal set (n=201)			External test set (n=15)		
	TCI (n=108)	non-TCI (n=93)	p value	TCI (n=7)	non-TCI (n=8)	p value
NCCLR	0.061 (0.029, 0.106)	0.068 (0.028, 0.115)	0.306	0.092 (0.032, 0.131)	0.073 (0.047, 0.113)	0.955
Protrusion			0.678			0.809
Absence	62 (57.40%)	57 (61.29%)		4 (57.14%)	3 (37.50%)	
Presence	46 (42.59%)	36 (38.71%)		3 (42.86%)	5 (62.50%)	

TCI, thyroid capsule invasion; NCCLR, the nodule-capsule contact length to perimeter ratio.

Table S4. The diagnostic performance of SVM was evaluated and compared under different expansion multiples of peri-tumor regions.

	α=1.1	α=1.2	α=1.3	α=1.4	α=1.5
Accuracy (95% CI)	0.695 (0.627, 0.763)	0.644 (0.574, 0.715)	0.746 (0.682, 0.810)	0.695 (0.627, 0.763)	0.689 (0.621, 0.757)
Sensitivity (95%CI)	0.694 (0.588, 0.801)	0.583 (0.469, 0.697)	0.778 (0.682, 0.874)	0.653 (0.543, 0.763)	0.694 (0.588, 0.801)
Specificity (95%CI)	0.695 (0.607, 0.783)	0.686 (0.597, 0.775)	0.724 (0.638, 0.809)	0.724 (0.638, 0.809)	0.686 (0.597, 0.775)
PPV (95% CI)	0.610 (0.504, 0.715)	0.560 (0.448, 0.672)	0.659 (0.558, 0.760)	0.618 (0.509, 0.728)	0.602 (0.497, 0.708)
NPV (95% CI)	0.768 (0.684, 0.853)	0.706 (0.617, 0.794)	0.826 (0.749, 0.904)	0.752 (0.668, 0.837)	0.766 (0.680, 0.852)
AUC (95% CI)	0.748 (0.684, 0.812)	0.708 (0.641, 0.775)	0.795 (0.735, 0.832)	0.770 (0.708, 0.832)	0.738 (0.673, 0.803)

CI, confidence interval; NPV, negative prediction value; PPV, positive prediction value; AUC, area under the curve.

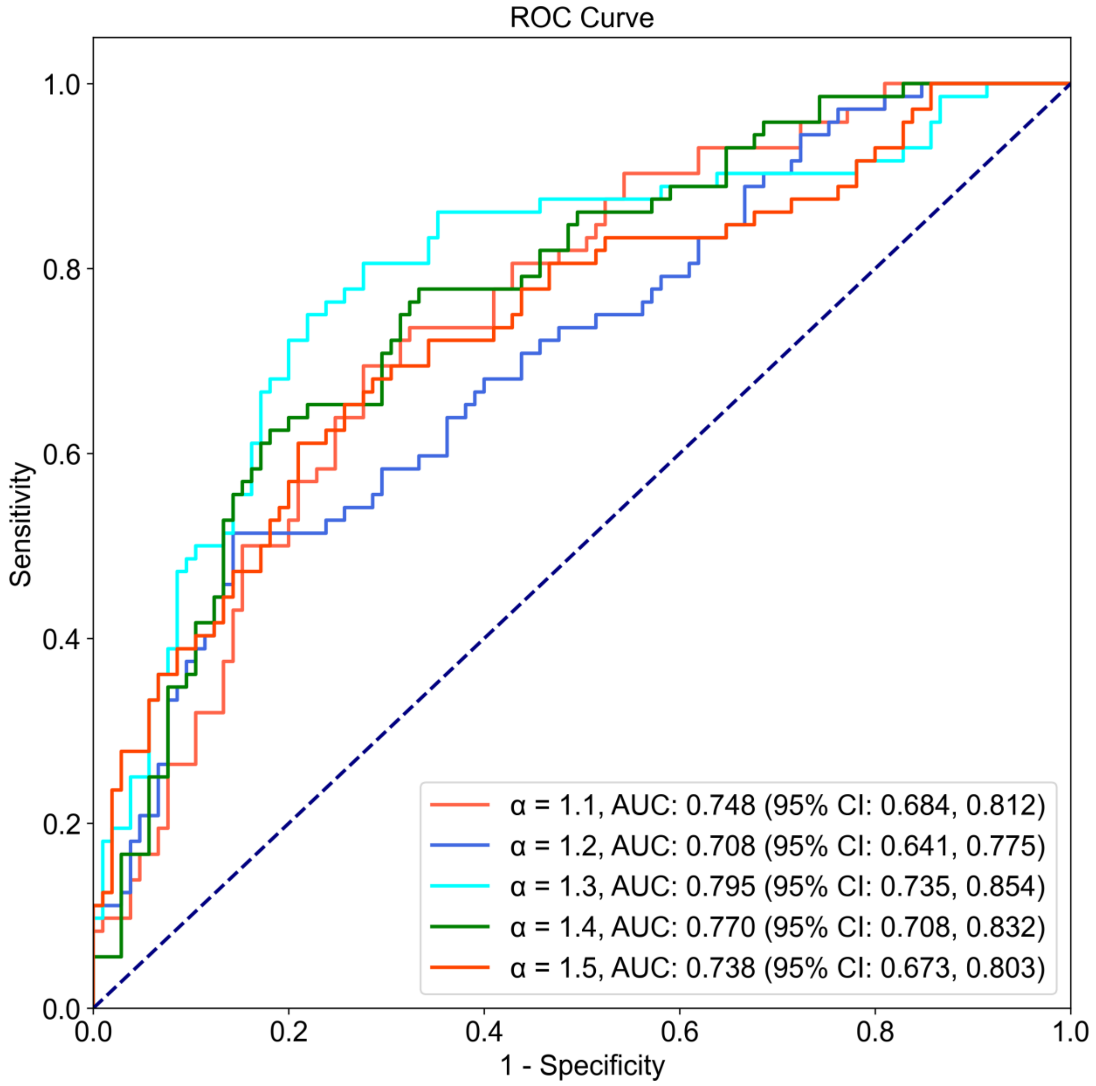

Figure. S1 Receiver operating curves of peri-tumor regions with different expansion multiple.

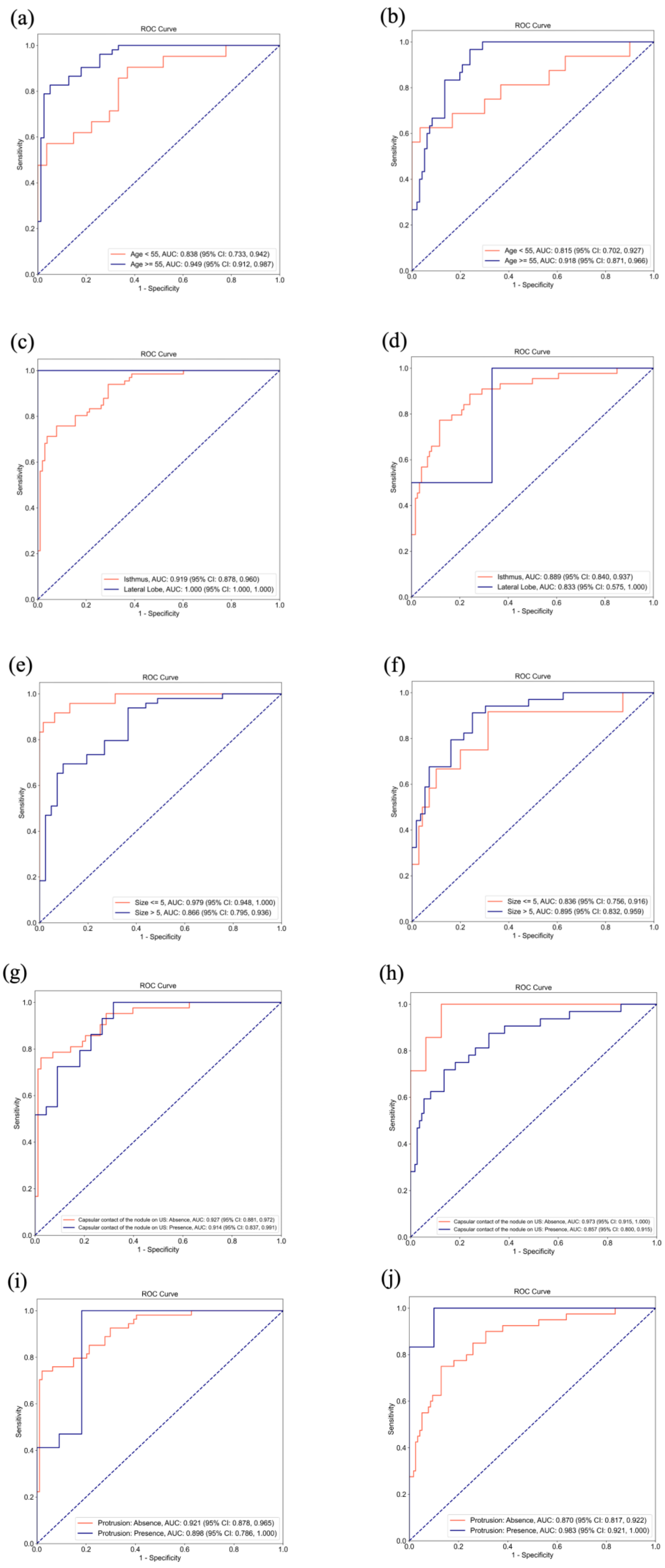
(a)
ROC Curve
Sensitivity
1 - Specificity
Age < 55, AUC: 0.838 (95% CI: 0.733, 0.942)
Age >= 55, AUC: 0.949 (95% CI: 0.912, 0.987)
(b)
ROC Curve
Sensitivity
1 - Specificity
Age < 55, AUC: 0.815 (95% CI: 0.702, 0.927)
Age >= 55, AUC: 0.918 (95% CI: 0.871, 0.966)
(c)
ROC Curve
Sensitivity
1 - Specificity
Isthmus, AUC: 0.919 (95% CI: 0.878, 0.960)
Lateral Lobe, AUC: 1.000 (95% CI: 1.000, 1.000)
(d)
ROC Curve
Sensitivity
1 - Specificity
Isthmus, AUC: 0.889 (95% CI: 0.840, 0.937)
Lateral Lobe, AUC: 0.833 (95% CI: 0.575, 1.000)
(e)
ROC Curve
Sensitivity
1 - Specificity
Size <= 5, AUC: 0.979 (95% CI: 0.948, 1.000)
Size > 5, AUC: 0.866 (95% CI: 0.795, 0.936)
(f)
ROC Curve
Sensitivity
1 - Specificity
Size <= 5, AUC: 0.836 (95% CI: 0.756, 0.916)
Size > 5, AUC: 0.895 (95% CI: 0.832, 0.959)
(g)
ROC Curve
Sensitivity
1 - Specificity
Capsular contact of the nodule on US: Absence, AUC: 0.927 (95% CI: 0.881, 0.972)
Capsular contact of the nodule on US: Presence, AUC: 0.914 (95% CI: 0.837, 0.991)
(h)
ROC Curve
Sensitivity
1 - Specificity
Capsular contact of the nodule on US: Absence, AUC: 0.973 (95% CI: 0.915, 1.000)
Capsular contact of the nodule on US: Presence, AUC: 0.857 (95% CI: 0.800, 0.915)
(i)
ROC Curve
Sensitivity
1 - Specificity
Protrusion: Absence, AUC: 0.921 (95% CI: 0.878, 0.965)
Protrusion: Presence, AUC: 0.898 (95% CI: 0.786, 1.000)
(j)
ROC Curve
Sensitivity
1 - Specificity
Protrusion: Absence, AUC: 0.870 (95% CI: 0.817, 0.922)
Protrusion: Presence, AUC: 0.983 (95% CI: 0.921, 1.000)

Figure. S2 The ROC curves of the AI model in different age subgroups on the internal test set (a) and external test set (b); The ROC curves of the AI model in different lacation subgroups on the internal test set (c) and external test set (d); ROC curves of the AI model in different lesion size subgroups on the internal test set (e) and external test set (f); The ROC curves of the AI model for the Capsular contact of the nodule on US subgroup on the internal test set (g) and external test set (h); The ROC curves of the AI model for the Protrusion subgroup on the internal test set (i) and external test set (j).

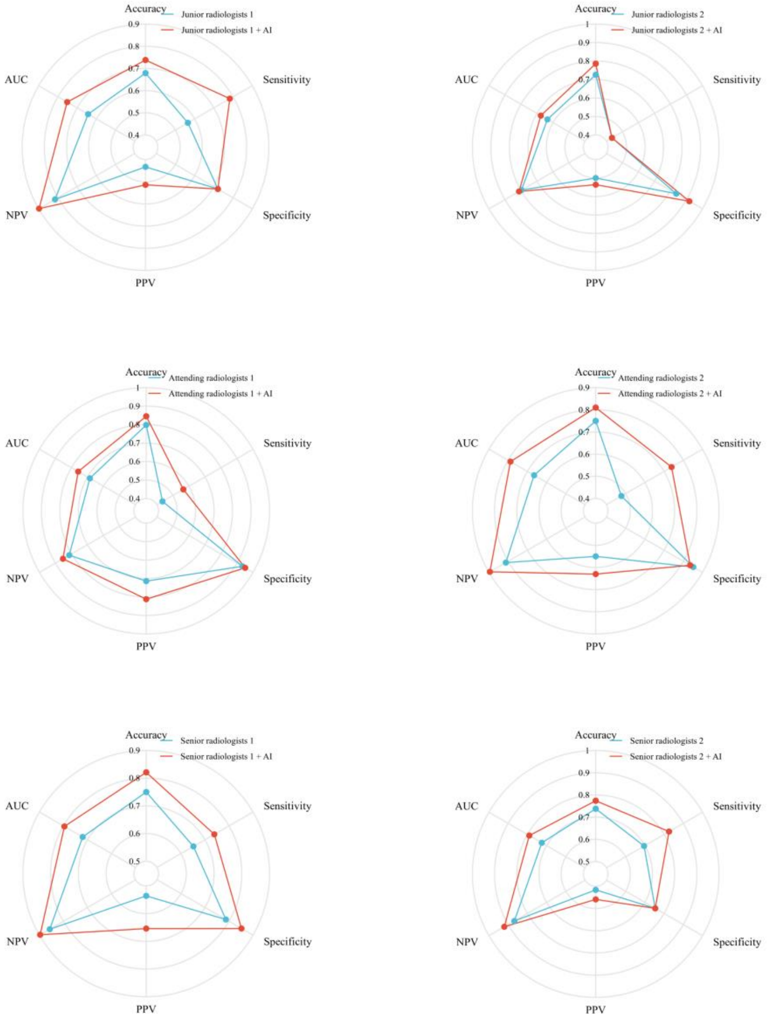

Figure. S3 Radar map illustrates the changes in diagnostic performance metrics among 6 radiologists with and without model assistance.

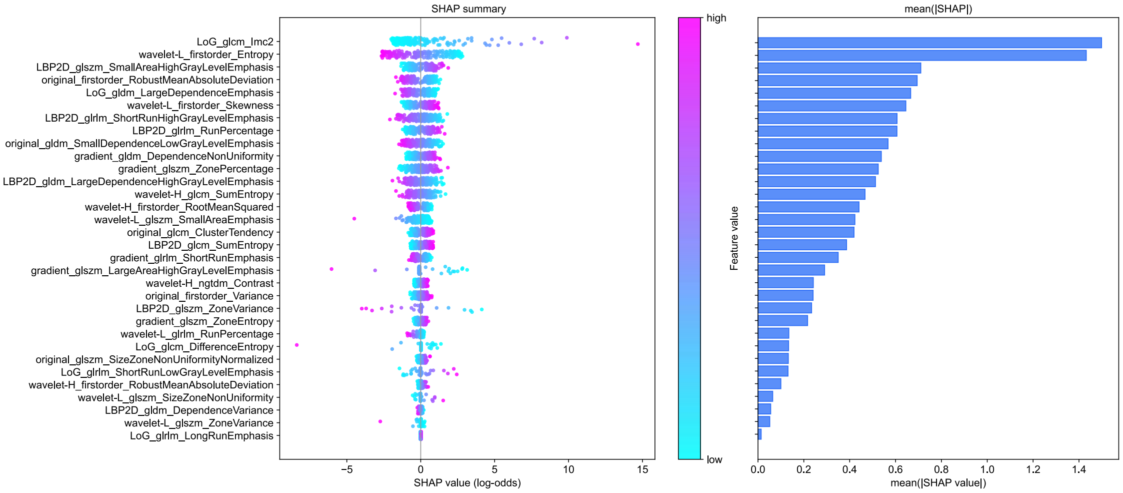

Figure. S4 SHAP-based feature importance visualization. (Left) Beeswarm plot showing the distribution of SHAP values for each feature across all samples in the internal test set, where each point represents an individual patient, the horizontal position reflects the SHAP value, and the color indicates the original feature value (red = high, blue = low). Features are ordered by their overall importance. (Right) Bar plot of the mean absolute SHAP values across all samples in the internal test set, representing the average contribution of each feature to the model predictions.

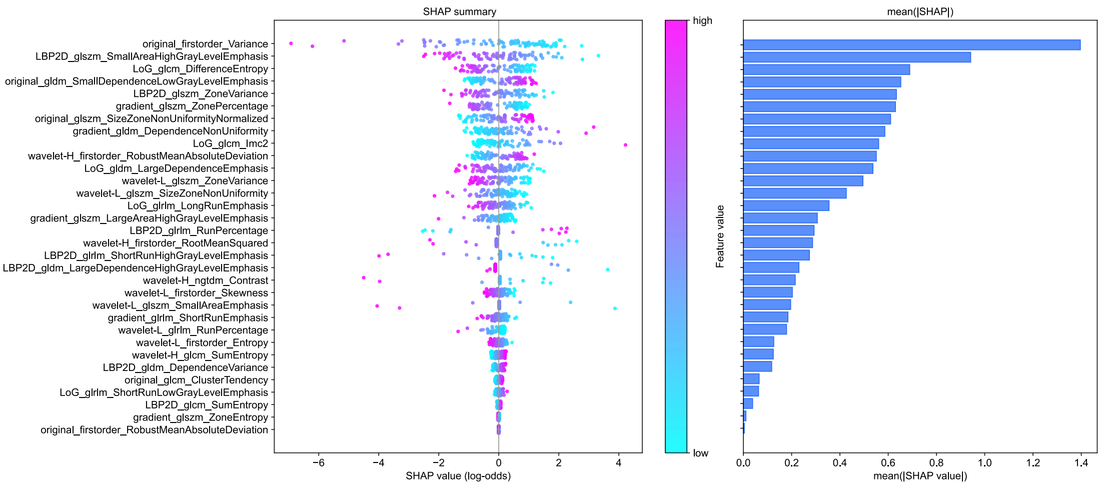

Figure. S5 SHAP-based feature importance visualization. (Left) Beeswarm plot showing the distribution of SHAP values for each feature across all samples in the external test set, where each point represents an individual patient, the horizontal position reflects the SHAP value, and the color indicates the original feature value (red = high, blue = low). Features are ordered by their overall importance. (Right) Bar plot of the mean absolute SHAP values across all samples in the external test set, representing the average contribution of each feature to the model predictions.
